# Stochastic Modeling for the Expression of a Gene Regulated by Competing Transcription Factors

**DOI:** 10.1371/journal.pone.0032376

**Published:** 2012-03-14

**Authors:** Hsih-Te Yang, Minoru S. H. Ko

**Affiliations:** Developmental Genomics and Aging Section, Laboratory of Genetics, National Institute on Aging, National Institutes of Health (NIH), Baltimore, Maryland, United States of America; University of Rome, Italy

## Abstract

It is widely accepted that gene expression regulation is a stochastic event. The common approach for its computer simulation requires detailed information on the interactions of individual molecules, which is often not available for the analyses of biological experiments. As an alternative approach, we employed a more intuitive model to simulate the experimental result, the Markov-chain model, in which a gene is regulated by activators and repressors, which bind the same site in a mutually exclusive manner. Our stochastic simulation in the presence of both activators and repressors predicted a Hill-coefficient of the dose-response curve closer to the experimentally observed value than the calculated value based on the simple additive effects of activators alone and repressors alone. The simulation also reproduced the heterogeneity of gene expression levels among individual cells observed by Fluorescence Activated Cell Sorting analysis. Therefore, our approach may help to apply stochastic simulations to broader experimental data.

## Introduction

It has been widely accepted that gene expression regulation follows a stochastic mechanism at the single gene or cell level [1], [2], [3], [4], [5], [6], [7], [8]. To predict the variability of a reporter gene's expression in numerical simulations, discrete stochastic (Markov jump process) models, e.g., the Gillespie algorithm [9] and Peccoud and Ycart model [10], and continuous stochastic model driven by chemical Langevin equation (CLE) [11] have been widely used [12]. However, Gillespie's algorithm and CLE are limited to modeling well-studied biological pathways [13], [14], [15], because they require the detailed chemical kinetics on interactions of individual molecules, which is often not available for the analyses of biological experiments. Similarly, the Peccoud and Ycart model require the measurement of the parameters for promoter state switching, mRNA burst (size and frequency), and mRNA degradation at the single molecule level in a single cell [1], [16], [17].

Due to these limitations, many biological systems have been modeled without using a stochastic simulation or have not been modeled. For example, Ferrell and Machelder have used the Hill Coefficient to model the conversion of continuous hormone stimuli to all-or-none responses by positive feedback regulation in cell signaling [18]. Similarly, Werner et al. have modeled the switch-like activity of the Epstein-Barr virus (EBV) C promoter, regulated by competitively binding two types of transcription factors (TFs) (one from the virus and the other from the host) without using stochastic simulation [19]. Perhaps one of the best examples of an experiment that does not use models was carried out by Rossi et al. [20]. They used a synthetic transcription unit with the overlapping promoter regions bound by either doxycycline (dox)-controlled activators alone, or repressors alone, or both [20]. The authors have demonstrated that depending on the concentration of inducer, dox, this dox-inducible system yields graded (rheostat) or all-or-none (on/off switch) responses at the transcriptional level even in the isogeneic cell population. The authors have extracted the Hill coefficients from Fluorescence Activated Cell Sorting (FACS) data and demonstrated that both activators and repressors compete for the same promoter. However, the Werner's approach [19] was unable to directly quantify the synergistic (Hill coefficient) and stochastic (cellular heterogeneity) characteristics of this synthetic transcription unit [20], because these experiments lack the kinetic rates and binding affinity constants of TFs.

**Figure 1:**
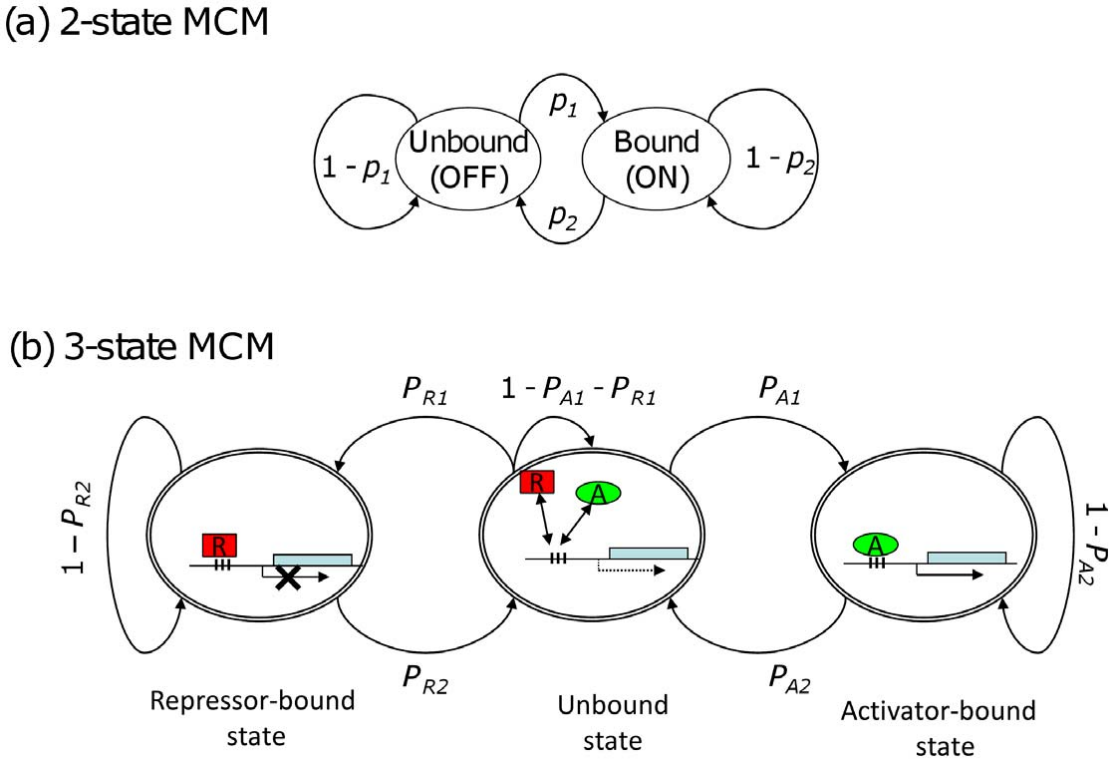
Transition diagrams for Markov-chain model (MCM). (a) A 2-state MCM, redrawn from the original Figure 2 in [21]. *p_1_* and *p_2_* are probabilities of transitions between a state of active transcription (ON), where TFs bind to a promoter and form a stable transcription initiation complex, and a state of no transcription (OFF). (b) 3-state MCM. To account for both activator-bound and repressor-bound states, two 2-state MCMs are combined, where unbound state (i.e., neither activator nor repressor bound) represents a state of basal-level transcription. *P_A1_*, *P_A2_*, *P_R1_* and *P_R2_* are transition probabilities between the states.

Accordingly, it is desirable to develop a method that allows stochastic simulation even if detailed information on the interaction of individual molecules is not available. To this end, we have used an intuitive approach, a Markov-Chain model (MCM) [21], which was initially formulated to simulate the stochastic behavior of a glucocorticoid hormone-inducible gene expression system [22] (Figure 1a). To simulate the experimental results by Rossi et al. [20], we extended the original 2-state MCM (TF-bound and TF-unbound) to a 3-state MCM (repressor-bound state, activator-bound state, and none-bound state). We have found that these MCMs can faithfully reproduce the observed cellular heterogeneity of a reporter gene observed by FACS experiments and also accurately predict a Hill-coefficient in the presence of both activators and repressors based on the experimental data obtained by activator only and repressor only trials. Our stochastic simulation can, thus, provide a new tool to explore the origins and controls for the stochasticity of gene regulatory networks by using simple dose-response data.

## Results

### Experimental data used for the analyses

Without a positive feedback loop in the signaling cascades [18], [23], [24], [25], [26], Rossi et al. have generated the switch-like or all-or-none patterns of gene expression at the transcriptional level, in which the activators and repressors compete for the same promoter regions of the reporter gene [20]. The authors have created three different cell lines: the presence of the activator (A) only, the presence of the repressor (R) only, and the presence of both activator and repressor (A+R). These cells have been used separately to produce dose-response curves ((dox concentration [dox]) vs. promoter activity presented as % maximum green fluorescent protein (GFP)) by adding different concentrations of dox in the cell culture medium (see the original Figure 2 in Rossi et al., 2000). They obtained the observed Hill coefficient from these dose-response curves: 1.6 for the presence of the activator only, 1.8 for the presence of the repressor only, and 3.2 for the presence of both the activator and repressor. The authors have suggested that both multiplication and addition of the Hill coefficients 1.6 and 1.8, as it has been done customarily, can produce 2.8 or 3.4, respectively, which are close to the observed Hill coefficient 3.2. One of our goals is to explain why the observed Hill coefficient (3.2) is different from the calculated Hill coefficient (2.8 or 3.4).

**Figure 2:**
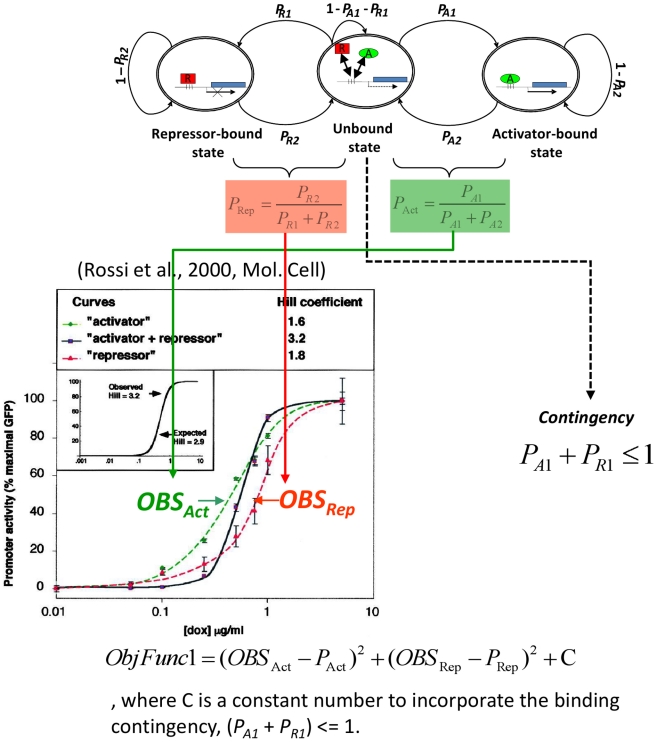
A strategy of parameter estimation for a 3-state MCM. All the parameters for the 3-state MCM were estimated from the published data only on the dose-response experiments of activator only and repressor only [20]. In the case of activator only, parameters in *P_Act_* (Eq. **3** or Eq. **10**) were estimated by using the observed dose-response curve (*OBS_Act_*: a Hill function of [dox]) represented by the equation (Eq. **4** or Eq. **11**) in the Materials and Methods section. The repressor only case (*OBS_Rep_*, *P*_Rep_) was handled in the same manner.

The only other available data from the experiments by Rossi et al. is the single-cell analysis of GFP expression by FACS (see the original Figure 3 in Rossi et al., 2000). By visually inspecting the distributions of GFP intensities measured by the FACS analyses, we obtained approximate peaks of intensities at 0.2 for the presence of the repressor only, 20 for the presence of the activator only, and 1.0 for the absence of either the activator or repressor.

**Figure 3:**
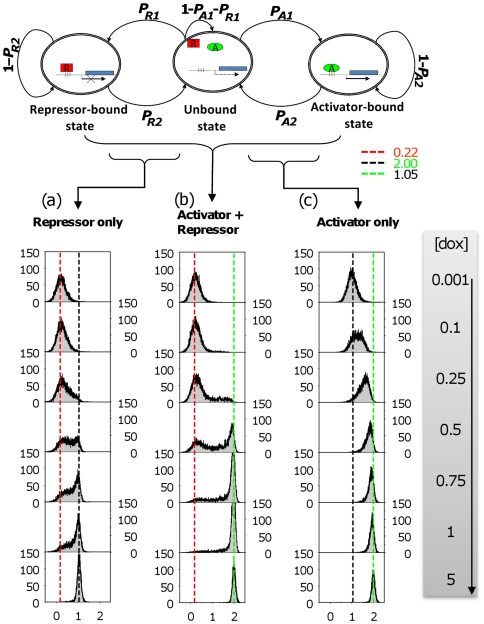
Stochastic simulation using a 3-state MCM yields all-or-none responses. In a cell population, steady-state distributions of gene induction were stochastically simulated by the 3-state MCM using the estimated parameters described in Figure 2. Red, black, and green lines present the peaks of transcription levels in the “repressor-bound,” “unbound,” and “activator-bound” states, respectively. A gray vertical bar indicates the increasing concentrations of [dox], which correspond to the experimental conditions reported in [20].

### Construction of a 3-state MCM

To model the experimental results, three different types of cell lines have to be considered: a GFP-tagged transcription cassette mediated by a dox-controlled repressor (R); a dox-controlled activator (A); and both (A+R). Both repressors only and activators only (the first two types [A or R]) can be directly formulated into the 2-state MCM (Figure S1a), in which the accessibility of promoter is based on the binding and unbinding states of a single transcription factor (TF). Modeling gene regulation by the presence of both the repressors and activators (the third type [A+R]) is not straightforward, but the 2-state MCM can be expanded to a 3-state MCM (Figure 1b) by assuming the state where neither activator nor repressor binds to the promoter, which produces the leaky basal level of gene expression. This “binding contingency” assumption can be justified, because the activator has to be unbound before the repressor can bind and *vice versa*.

### Parameters estimated from the dose-response experiments of activator only and repressor only

To apply the 3-state MCM to the experimental results by Rossi et al. [20], we proposed a new way to estimate these parameters from dose ([dox])–response (GFP) curves, which can be fitted with the Hill function (GRF, see the Method section). Because the binding affinity of the activator and repressor to a tet operator (tetO) is highly regulated by dox, the switching probabilities of MCM are assumed to be varied with respect to [dox] and reasonably defined as the Hill function of [dox] (Eq. **9**).

The 3-state MCM (Figure 1b) consists of four switching probabilities (*P_A1_*, *P_A2_*, *P_R1_* and *P_R2_*). By assuming the same physical and chemical properties of the activator or repressor itself in the three types of cell population (A, R, and A+R), the 3-state MCM dose-response curves of activator (*P_Act_*) and repressor (*P_Rep_*) still keep the same sigmoidal flexure as those obtained experimentally for activators alone (*OBS_Act_*) and repressors alone (*OBS_Rep_*) (Figure 2). This notion leads to the derivation of an objective function (Eq. **12** or **13**) that is used to optimally minimize the differences between *P_Act_* and *OBS_Act_* together with *P_Rep_* and *OBS_Rep_* (Figure 2 and -method) under the assumption of “binding contingency”. After optimizing this objective function, these switching probabilities can be estimated as the Hill function of [dox] and plotted as dose (dox) – response (probability) curves (see the following sections for details). Therefore, the four switching probabilities were varied with respect to different levels of [dox].

### Stochastic simulation yields gradual or switch-like responses

By plugging the estimated switching probabilities into the 3-state MCM for stochastic simulation (Eq. **14**), our model produced the steady-state responses of 10,000 individual runs in the isogenic cell population, in which no cell-to-cell interaction is assumed. Consistent with the FACS data [20], the simulation results not only exhibited graded patterns (Figure S1b) for activator or repressor alone by a 2-stateMCM, but also manifested all-or-none patterns of gene expression (Figure 3b) in the presence of both activator and repressor at the optimal conditions ([dox] = 2.5∼7.5 µg/ml) by the 3-state MCM. The generation of this switch-like gene expression pattern implies that the “binding contingency” between activators and repressors (i.e., the notion that both activators and repressors cannot bind to the regulatory regions at the same time) is compatible with the conclusion that the competition of TFs (A+R) for the same DNA regulatory element is required and sufficient for all-or-none responses [20].

### Model prediction matches more closely to the experimental observation

**Figure 4:**
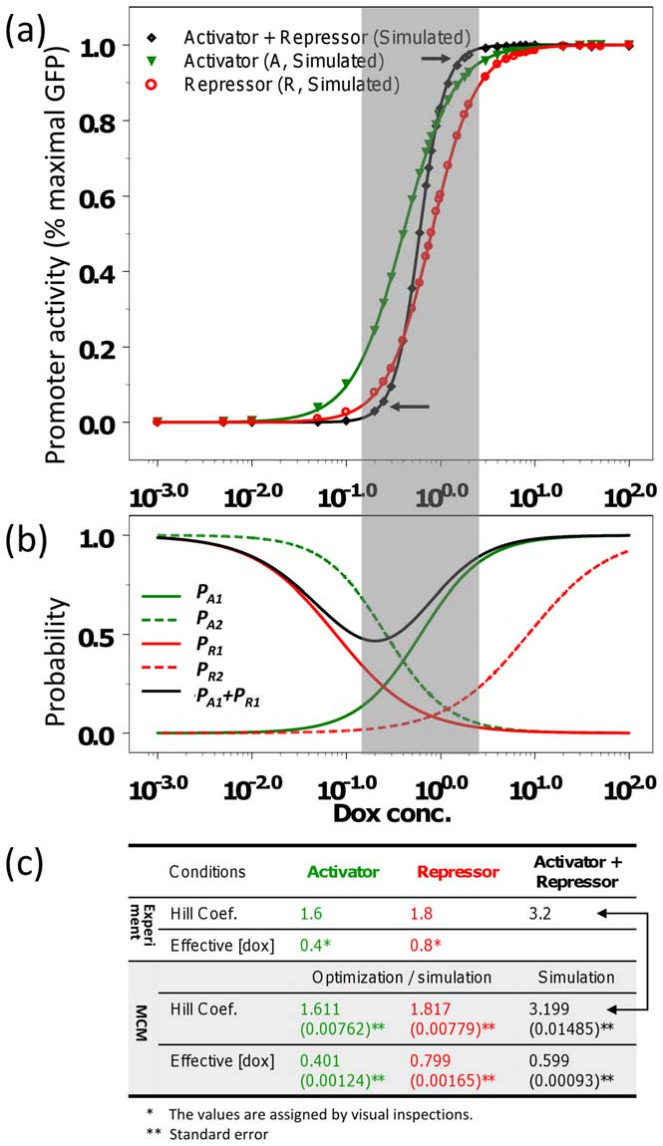
Model prediction matches more closely to the experimental observation. (a) Simulated dose-response relationship between [dox] and promoter activity (normalized gene induction levels). (b) Hill functions showing estimated switching probabilities (*P_A1_, P_A2_, P_R1_,* and *P_R2_*) against [dox]. Values of P_A1_+P_R1_ against [dox] are also shown. To show the relationship between (a) and (b), these graphs are aligned by the [dox]. (c) Comparisons between model predictions and experimental observations.

To generate an ensemble Hill coefficient from total population responses in the 3-state MCM in the steady state, we found that the 7 [dox] conditions simulated in Figure 3 were not sufficient. We therefore carried out more extensive stochastic simulations and increased the number of [dox] conditions to 34 for the activator only and repressor only conditions, and averaged them to plot the dose-response relationship between [dox] and normalized promoter activities (Figure 4a). As expected, the dose-response relationship followed a sigmoidal curve. Although the parameter optimizations for the dose-response experiments were carried out to have the Hill coefficients for activator alone or repressor alone be close to 1.6 or 1.8 (numbers in green and red in Figure 4c), the results indicate that the 3-state MCM can retain the dose-response characteristics of either activator alone (1.6) or repressor (1.8) alone.

We next carried out extensive stochastic simulations in the presence of both activator and repressor for 34 [dox] conditions and plotted the dose-response relationship. Data points closely followed the dose-response curve (Figure 4a, a curve in black). Although all stochastic simulations for activator only, repressor only, and both activator and repressor (Figure 3 and Figure 4a) were generated under the same values of switching probabilities (*P_A1_*, *P_A2_*, *P_R1_* and *P_R2_*, green and red lines in Figure 4b), the dose-response curves derived from the activator/repressor conditions (black line in Figure 4a) was steeper than those of the activator only or repressor only (green and red lines Figure 4a). This implies that the binding contingency between activators and repressors may lead to more sensitive and cooperative gene induction than that mediated by either activator alone or repressor alone. By deducing the Hill function from the dose-response curves (Figure 4a), a Hill coefficient for the presence of both activator and repressor was calculated to be 3.2 (numbers in black and arrows in Figure 4c). This number was indeed very close to the experimentally observed Hill coefficient for the presence of both activator and repressor.

To address how these switching probabilities relate to the synergistic or cooperative responses of gene induction, we focused on the critical ranges (gray region in Figure 4a) and found that the switching probabilities (red lines in Figure 4b) of repressor alone (R) are lower than those (green lines in Figure 4b) of activator alone (A). Moreover, not only the 3-state MCM (Figure 3b) for both TFs (A+R) but also the 2-state MCM for repressor alone (Figure 3a) manifested switch-like patterns of stochastic gene expression. This implies that the repressor itself, rather than activator itself, may possess the pivotal role of having all-or-none patterns of stochastic gene expression for the third type of cell population (A+R) in the experiments by Rossi et al.

## Discussion

By using the MCM and estimating its parameters from dose-response experiments of either repressor alone or activator alone, our modeling is able to predict the stochasticity and cooperativity of gene induction experiments in the presence of both activators and repressors [20]. The MCM approach is in sharp contrast to the conventional approach, i.e. the Gillespie algorithm [9] and the Peccoud and Ycart model [10], in the following ways.

First, the detailed molecular reactions in the genetic constructs of experiments by Rossi et al. may encompass over twelve kinetic rate constants required for computer modeling, such as TF dimerization, dox conjugating to repressor/activator protein and TF binding/unbinding to a promoter with multiple binding sites. If these rate constants are available and experimentally tested, the model equations can be formulated for the Gillespie's algorithm using mass action rules and stochastic simulations. In contrast, only four switching probabilities, which represent four Hill functions of [dox] with eight parameters, are required for constructing a 3-state MCM without the kinetics of molecular interactions. Even Peccoud and Ycart's model is able to simplify such gene induction processes by introducing the rates of gene switching and mRNA biosynthesis, although the parameter estimations highly rely on single-molecule experiments. Second, only cell population-averaged dose-response curves at the steady state measured by FACS are affordable. The parameter values of the conventional methods were unable to be directly extracted from simple dose-response experiments. Consequently, our stochastic simulation of the 3-state MCM can be used to precisely predict the Hill coefficient of gene induction measured by regular biological experiments.

Recently, Kim and O'Shea mathematically established a thermodynamics model to fit the dose-response gene expression of the *PHO5* promoter from a single yeast cell by optimally searching a set of parameter values, which can be used to explain the different dynamics of gene induction among *PHO5* promoter variants. The genetic constructs and relevant designs in this experiment are very similar to those in the experiments by Rossi et al. Thus, we applied this model to the 3-state gene induction of experiments by Rossi et al. (File S1). As shown in Table 1, the Hill coefficient predicted by the thermodynamic model is farther from the experimentally observed value than that predicted by the 3-state MCM simulation. This indicates that the model by Kim and O'Shea cannot be directly applied to experimental results by Rossi et al.

**Table 1:** Comparisons of the predicted parameters of Hill function for gene induction mediated by two competing factors (A+R).

	Conventional(a)	Conventional(b)	3-state MCM(c)	Calculated(d)	Observed(e)
Hill coefficient	2.567 (0.00256)*	2.692 (0.00196)*	3.199 (0.01485)*	3.4 or 2.88	3.2
Effective [dox]	0.699 (0.00031)*	0.483 (0.00015)*	0.599 (0.00093)*		

(a) *P^s^*_Act_: The transcriptionally active promoter is defined by only activator-bound state.

(b) *P^s^*_Act_+*P^s^*_Unb_: The transcriptionally active promoter is defined by both unbound and activator-bound states that are the summation of constitutively and fully expressed gene.

(c) Our method reported in this paper.

(d) Addition (1.6+1.8) or multiplication (1.6×1.8) as reported in Rossi et al. (2000).

(e) An experimentally observed value reported in Rossi et al. (2000).

* Standard error.

In general, besides the randomness of basal levels and mRNA degradations in Eq. **5**, the size of a time step (*Δt*) is a critical factor that affects the stochasticity or randomness of the simulation. In the Gillespie algorithm, the time step sizes are varied in relation to the total amount of rate changes and molecular numbers in the whole dynamic system. In the stochastic simulation by MCM, the step size is fixed so that the majority of cellular variability may arise from the switching back and forth between “ON” and “OFF” states. However, to enhance the numerical integration, the stochastic differential equation, i.e. Ito integration, together with variable time steps could also be incorporated into MCM stochastic simulations.

One important aspect of the MCM is its ability to produce both the graded or all-or-none patterns of gene expression by changing *p_1_*, which corresponds to the concentration of a TF and *p_2_*, which corresponds to the stability of the TF-binding to the enhancer/promoter (i.e., transcription initiation complex) [21]. It is worth commenting here on the MCM and all-or-none patterns of gene expression, because such an all-or-none pattern of stochastic gene expression has been found to be a major molecular basis for cell fate determination [7], [29]. A recent work by To and Maheshri has demonstrated that high turn-over rates and multiple DNA binding elements of TFs can induce all-or-none responses in the synthetic positive feedback system in the steady state without having bistability itself [16]. We found that the MCM can also handle this case by assuming that *p_1_* and *p_2_* are correlated to the duration of TF presence and the number of TF binding sites, respectively. By searching the *p_1_* and *p_2_* space by simulations, one can find the *p_1_* and *p_2_* probabilities that produce the all-or-none gene expression patterns [3], [21]. Another important point of the MCM is its ability to examine the time-course of the gene expression status in individual cells, as we show examples of dynamical fluctuations over time in two individual cells, which manifest either graded or all-or-none patterns of gene expression at the steady state (Figure S2). By examining many cells in the population in this manner, the MCM approach can provide a comprehensive way to depict different types of cellular heterogeneity for gene induction.

A stochastic simulation of a 3-state MCM for activator-repressor controlled gene induction is easily performed by experimental biologists due to three points: (a) mapping the gene induction processes to a Markov chain model only requires logical thinking; (b) the parameter values are estimated from simple dose-response experiments; (c) the Hill coefficient can be predicted by stochastic simulation rather than by deriving a dimensionless analytical solution from a set of complicated ODEs.

Finally, we believe that the approach we have demonstrated here can be easily applied to the stochastic simulation of many other biological systems, including signaling and metabolic pathways, because the implementation of the approach is intuitive and does not require training in advanced physics and chemistry.

## Materials and Methods

### 2-state MCM

#### Design principles of the MCM for gene induction

Chromatin structures (i.e. histone modifications and nucleosomal remodeling) [27], [30], [31] and TF-binding to enhancer/promoter regions [21], [32], [33], [34] have been known to significantly modulate transcription initiation in eukaryotic genes. By assuming rate-limiting steps among these molecular processes [21], [34], we regarded the state of the enhancer/promoter of gene induction as either the “ON” or “OFF” state, in which the TFs bind or unbind (Figure 1a). Once the promoter is bound by TFs (activators), the gene becomes transcriptionally active and produces a fixed quantity of mRNAs by iteratively loading and releasing RNA polymerase per unit time, otherwise the gene is silenced or inactive with no production of mRNA transcripts. Every unit time, the system follows a transition diagram [21] (Figure 1a), in which the stochastic transitions between “OFF” and “ON” states of the gene enhancer/promoter are controlled by two parameters: *p_1_* is the probability of switching from the “OFF” to “ON” state to form stable transcription initiation machinery, resulting in the synthesis of mRNA molecules, whereas *p_2_* is the probability of dissociating the transcription complexes to shut down gene expression. The system remains in the same state at the probabilities of (1−*p_1_*) and (1−*p_2_*), respectively. After obtaining parameter values (*p_1_* and *p_2_*) and model simulations, gene induction can be represented as telegraphs (Figure S2b), in which the states of enhancer/promoter activity are discretely changing over time, resulting in the accumulation of mRNAs, which are also degraded at a fixed rate.

Probabilities *p_1_* and *p_2_* can be considered independent, as *p_1_* is correlated to the concentration of TFs, and *p_2_* is the probability of dissociation of the TF complexes on the enhancer/promoter regions, which should be independent of the concentration of TFs [21], [32], [35]. However, in this paper we have also considered the case with *p_2_* = 1−*p_1_*, in which *p_2_* is dependent on the *p_1_*.

#### Properties of the MCM at steady state

Based on the Markov chain and the schematics of gene induction (Figure 1a), the likelihood of a both “ON” and “OFF” state (*P_ON_* and *P_OFF_*) of enhancer/promoter activity can be formulated by the forward and reverse switching probabilities (*p_1_* and *p_2_*) with respect to time evolutions. The current state likelihood (t = *n*) of gene induction is determined by both the previous state (t = *n*−1) and the switching probabilities (*p_1_* and *p_2_*). *P_ON_* (*P_OFF_*) is the summation of the probabilities to maintain its original state and to transition from the “OFF” (“ON”) state. Consequently, the likelihood of the “ON” and “OFF” state (*P_ON_* and *P_OFF_*) is always changing with time.
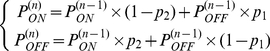
(1)When this dynamical system reaches to the steady state, *P_ON_* ^(*n*)^ and *P_OFF_* ^(*n*)^ will converge to dimensionless *P_ON_* and *P_OFF_*. Then at the steady state Eq. **1** becomes:
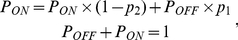
(2)where the summation of “ON” and “OFF” state likelihood is equal to 1. By solving Eq. **2**, the analytical solutions of state likelihood are obtained at the steady state as the function of switching probabilities (*p_1_* and *p_2_*), which are the parameters that will be estimated from the experimental data (see the next section):
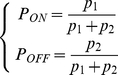
(3)


#### Parameter estimations

Gene-regulatory function (GRF) is proposed to quantify promoter activity or gene expression by formulating the non-linear function of TF concentration [27], [28], [36]. In general, GRF is experimentally measured as a sigmoidal dose-response curve, which can be mathematically expressed as the Hill function (Eq. **4**), whose parameters are TF binding affinity (*K_M_*), effective concentration to half-activated induction, and synergistic effect (*H,* Hill coefficient). Because our stochastic model simulates gene induction as the results of a telegraph (e.g. Figure S2b) based on switching probabilities (*p_1_* and *p_2_*), the parameters (switching probabilities) must be directly connected to the GRF based on the experimental results. To this end, we converted the GRF or Hill function into the probabilistic models as follows. The promoter activity is proportional to the fractional binding of the TF on the target gene. In other words, the percentage or occupancy of the promoter bound by transcriptional activator can be defined as the switching probability from the “OFF” to “ON” state of enhancer/promoter accessibility. Note that all above assumptions regarding promoter activity are based on multiple copies of the target gene in the cell population, whereas the switching probabilities (*p_1_* and *p_2_*) are the stochastic model for the induction of a single gene (one DNA template) in an individual cell [21], [22].
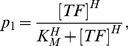
(4)where [*TF*] is the concentration of TF and input of GRF. *p_1_* is the output of GRF, defined as the switching probability (0∼1) by promoting the “OFF” to “ON” state of enhancer/promoter.

#### Numerical solver for stochastic simulation

The molecule number of each mRNA species (*X*) in a single cell is dynamically changed over time by both synthesis (birth) and degradation (death). The kinetic rate equation for the turnover of RNA molecules is generally expressed and integrated as follows:
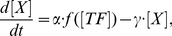
(5)where *α* is the rate of gene transcription to synthesize mRNA molecules and *γ* is the first-order degradation rate of mRNAs. *Δt* is the unit of time interval for numerical integration. We took the following approach to convert the deterministic system into a stochastic process,
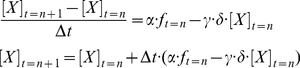
(6)Based on our previous study [21], this equation is slightly modified by putting the two random effects (Eqs. **5** and **6**) into “birth” and “death” terms separately. The first effect is the state of enhancer/promoter accessibility (*f*_t = n_), which is highly dependent on the previous state (*f*_t = n−1_) and switching probability (*p_1_* or *p_2_*):
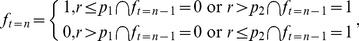
(7)where *r* is randomly selected from continuous numbers of uniform distribution within the range (0∼1). “1” indicates that a gene is activated to synthesize mRNAs with respect to the rate of transcription (*α*), whereas “0” represents that a gene is repressed and produces no RNAs during the time interval (from t = n−1 to t = n). The *p_1_* and *p_2_* values, which are the functions of [TF], the concentration of TF (Eq. **4**), may change over time series, if [TF] varies with time. The second effect (δ) is the factor of natural noise to interfere with the rate of mRNA degradation and is from normal distribution *N*(1, 0.5^2^).

#### Stochastic simulation for single gene induction

To apply a 2-state MCM to single gene induction, we adopted the solver (Eq. **6**).
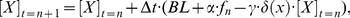
(8)where *BL*, equal to *γ*10*^δ^* ^(y)^, is the basal expression level including background noise (arbitrary unit) presented in the FACS result and the *μ* of δ(y) (the second effect in Eq. **6**) equals to *N*(0, 0.5^2^). This additional term (*BL*) was incorporated into the simulation to model the basal level of repressor-mediated (“R” condition) gene induction measured at [dox] = 0 by FACS. The time step size (Δ*t*) is assumed to be 1. We used 2.0 and 0.2 as the rate of transcription (alpha, α) and mRNA degradation (gamma, γ), respectively. Although these are arbitrary values, at least they are similar to the kinetic parameters of GFP mRNA biosynthesis in yeast [34]. Furthermore, the precise parameter values are, in general, not critical for this simulation, because these parameters mainly affect the steady-state level of gene expression, which is normalized to the range between 0 (0%) to 1 (100%), when the Hill coefficient and effective [dox] concentration are estimated from dose-response curves (Figure 4a and 4c). This normalization is necessary to compare our simulation results to the experimental results by Rossi et al. [20], as they have presented their results after such normalization in their paper.

We recorded the final outcomes of integrations of the single gene induction solver (Eq. **8**) at the steady state (t = 200 arbitrarily unit). This time point was chosen, because time evolutions (starting from t = 0) of [X] for three different [dox] conditions show that the mean value of [X] reaches the steady state (though minor stochastic fluctuation can still be seen) after 50 time cycles (Figure S3).

### 3-state MCM

The 3-state MCM is essentially the same as the 2-state MCM, but it follows two successive transitions of states: for gene activation, from Repressor-bound state to unbound state, and to Activator-bound state; for gene repression, from the Activator-bound state to unbound state, and to repressor-bound state (Figure 1b). Therefore, the 3-state MCM uses the same “Design principles of the MCM for gene induction” and “Properties of the MCM at steady state” as those described above in the 2-state MCM. The 3-state-specific methods are described below.

#### Parameter estimations

As depicted in the main text (Figure 1b), 3-state MCMs are driven by two forward (*P_A1_* and *P_R2_*) and two backward (*P_A2_* and *P_R1_*) switching probabilities. According to Eq. **4** of Methods, we assume these four switching probabilities are the Hill functions of [dox].
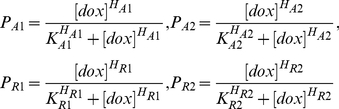
(9)In other words, these four switching probabilities of 3-state MCM are changed with different levels of [dox].

To obtain the above 8 parameters in the 4 Hill functions (4 switching probabilities), we can employ the analytical solutions (Eq. **3**) of state likelihood for the probability of attaining the “Activator-bound state (*P*_Act_)” and the “Repressor-bound state (*P*_Rep_)” from the “unbound state”:
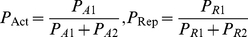
(10)In the same way, we can also obtain the observed probability of the “Activator-bound state (*OBS*_Act_)” and the “Repressor-bound state (*OBS*_Rep_)” based on the dose-response curves from the averaged cell population of FACS experiments [20]:
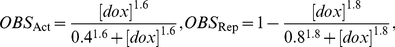
(11)where the values of the Hill coefficient and effective [dox] are adopted from the table in Figure 4c. To minimize the differences between the model and the experiment, the equations (Eqs. **12** and **13**) are organized into two types of objective functions: when gene induction is modeled by 3-state MCM.
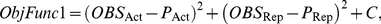
(12)where *C* is the “penalty” by setting 10,000 if the switching probabilities are not compatible with the assumption regarding the “binding contingency” between activator and repressor, (*P_A1_*+*P_R1_*)<1, or 0. when gene expression is characterized by 2-state MCM for repressor only or activator only (i.e., no binding contingency term is appended to the objective function),
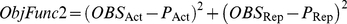
(13)After minimizing objective functions using MATLAB and the genetic algorithm (GA) toolbox v1.2 [37], four pairs of Hill function parameters are obtained (Table S1) and then plugged into four Hill functions (Eq. **9**) of switching probabilities (*P_A1_*, *P_A2_*, *P_R1_* and *P_R2_* in the 3-state MCM (Figure 1b)) to plot the sigmoid curves of [dox] in the Figure 4b. The other parameters are used to obtain the four switching probabilities (*P′_A1_*, *P′_A2_*, *P′_R1_* and *P′_R2_*) for stochastic simulations of 2-state MCM in the presence of repressor only or activator only from the experiments by Rossi et al. (Figure S1).

#### Stochastic simulation of 3-state MCM

Because the same HRIgfphGH bicistronic reporter is used for the three experimental conditions, i.e., “A”, “R” and “A+R”, [20], we used the same dynamical equation (Eq. **8**) and the corresponding parameters for stochastic simulation of both 2-state and 3-state MCM. The major difference between them is the function (*f*_t = n_, Eq. **7** v.s. Eq. **14**) of state transition regarding enhancer/promoter accessibility. As shown in Figure 1b, the state transitions in the 3-state MCM should proceed by two successive steps or “jumps” against the corresponding switching probabilities. Namely, these two successive steps can avoid a higher or over occurrence of the “unbound state”, which is the essential point to be passed through when the previous state is “activator-bound” or “repressor-bound” by a one-step move. In addition, there is no direct switching between repressive and active states in the 3-state MCM.

If the model reaches the “repressor-bound state” (“unbound” and “activator-bound” states), the promoter activity, *f*([TF]), is set to 0 (1 and 10). For the 3-state of MCM mediated by two forward (*P_A1_* and *P_R2_*) and two backward (*P_A2_* and *P_R1_*) switching probabilities, *f*_t = n_ can be expressed as:
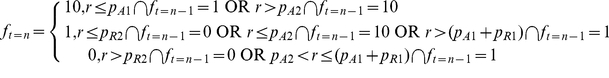
(14)


#### Dynamical fluctuations of individual cell at graded or all-or-none responses

To explore the underlying mechanisms for grade (Figure S1) and all-or-none (Figure 3b) responses regulated by activator alone (A) or repressor alone (R) and both (A+R), we carry out the dynamical fluctuations of single gene induction in two individual cells at the steady state and [dox] = 0.5 µg/ml by the general and 3-state MCM separately (Figure S2). Because the MCM is composed of digital and analog features, we aligned the telegraphs (i.e. enhancer/promoter accessibility, Figure S2a) with dynamical trajectories (i.e. accumulations of mRNA/protein, Figure S2b) to study the kinetics of promoter states for stochastic gene expression. In the graded mode of gene expression, the two 2-state telegraphs indicate that the switching back and forth between two of three states appears to be a random walk. However, in the all-or-none mode, the 3-state telegraph specifically illustrates that the enhancer/promoter tends to be stabilized at either the repressor-bound or activator-bound state. As time goes by for the all-or-none mode of gene induction, a reporter gene of the/a single cell which continuously expresses at a high level (“ON” state) will dramatically decrease to a low expression level (“OFF” state) for a period of time and then suddenly rise back and so on. Through this integrative view of digital and analog profiles (Figure S2), the dynamical fluctuations of simulated trajectories become more traceable and readable to aid in the understanding of molecular events for stochastic gene expression.

#### Plotting steady-state distribution of gene induction in a cell population

10,000 individual runs of the single gene induction solver were sequentially computed on the same computer platform with the same parameter values, except for the random numbers generated from the normal distribution (“norm” function in R) and uniform distribution (“runif” function in R). Steady-state outputs of 10,000 individual runs were recorded at the last observed time point (t = 200), averaged, calculated for the standard deviation (SD), and plotted by the high-density line plot of S-PLUS.

#### Statistics software used for this study

Most of the stochastic simulation solvers and scripts for statistical analyses are implemented by the R-2.11 language (http://www.r-project.org/). Figures for the stochasticity of single-cell populations and fitness of dose-response curves are plotted and performed by S-PLUS-8.0. Parameter estimations are done by MATLAB-2010a.

## Supporting Information

Figure S1 **Construction and stochastic simulation for 2-state MCM of repressor alone and activator alone.** (a) Two 2-state MCMs. One is the gene induction for activator only by switching forth and back between activator-bound and unbound states; the other is for repressor only with forward and reverse transitions between repressor-bound and unbound states. The red rectangle is the repressor and green oval is the activator. Note that the four switching probabilities (*P′_A1_*, *P′_A2_*, *P′_R1_* and *P′_R2_*) are different from the previous ones (*P_A1_*, *P_A2_*, *P_R1_* and *P_R2_*) in the 3-state MCM. (b) Stochastic simulation for cell population at the steady state.
(TIF)

Figure S2 **Dynamical fluctuations of simulated trajectories by MCM.** (a) At the steady state and [dox] = 0.5 µg/ml, two time-series trajectories of two “single cell” stochastic simulations, randomly selected from 10,000 individual computer runs. (b) The corresponding telegraphs. Under this condition, the stochastic simulations of cell population exhibit switch-like patterns by the 3-state MCM (Figure 3b) or graded responses by the 2-state MCM (Figure S1). Three different types of horizontal red lines are drawn to denote the three states of transcription levels.
(TIF)

Figure S3 **Averaged dynamical fluctuations of 10000 simulated trajectories by 3-state MCM.** Simulation was carried out in three different [dox] conditions. The duration of this stochastic simulation is set from 0 to 201 time cycles.
(TIF)

File S1 **Details about the modeling of experimental results reported in Rossi et al. (2000) by conventional method.**
(DOC)

Table S1 **Estimated parameter values.**
(DOC)
