# Supplementary figures and images for: Stochastic Modeling for the Expression of a Gene Regulated by Competing Transcription Factors
Source: PLoS One. 2012 Mar 14;7(3):e32376. doi: 10.1371/journal.pone.0032376 (PMC3303788; doi:10.1371/journal.pone.0032376)

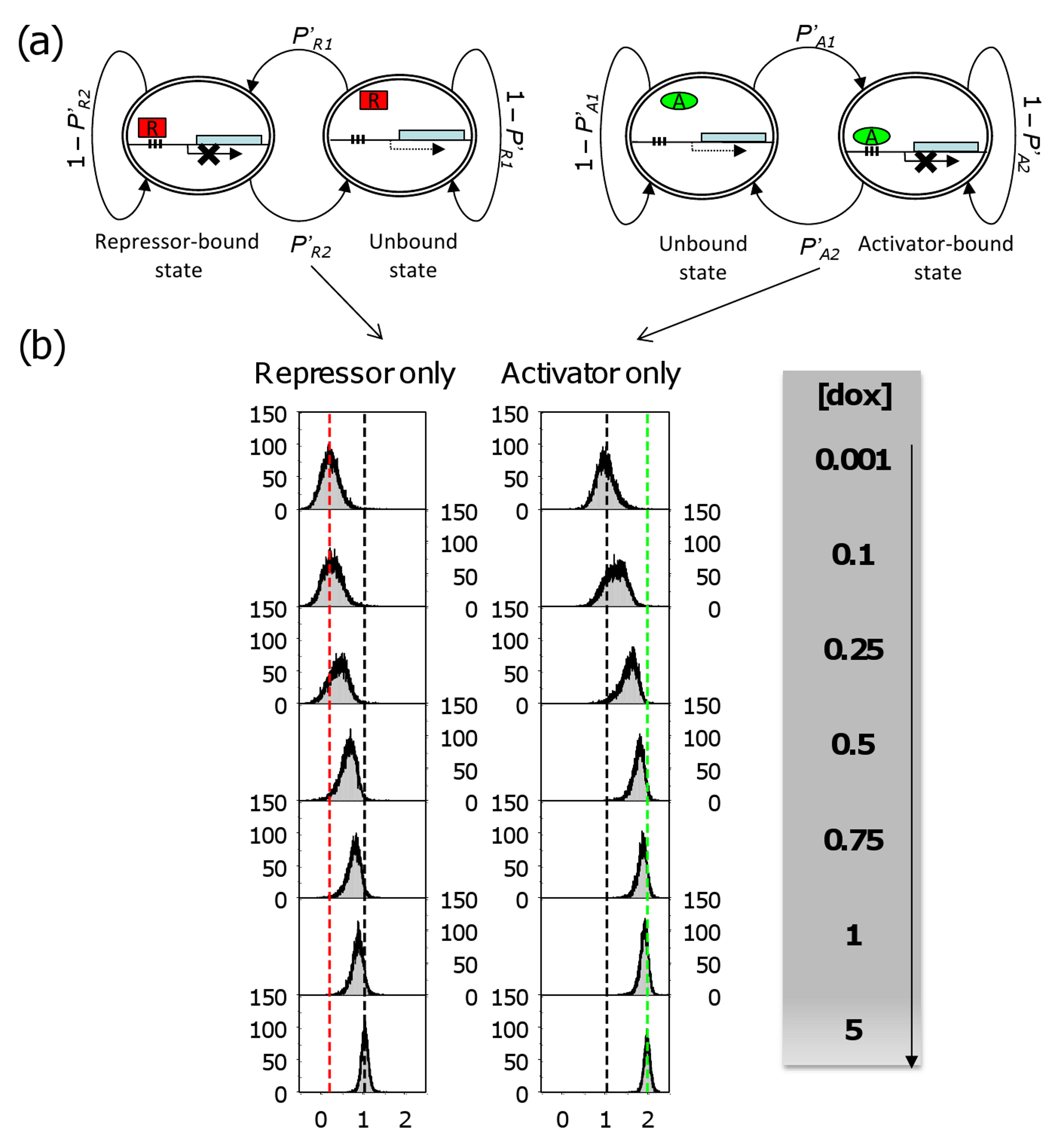

Supplement: Figure S1 — Construction and stochastic simulation for 2-state MCM of repressor alone and activator alone. (a) Two 2-state MCMs. One is the gene induction for activator only by switching forth and back between activator-bound and unbound states; the other is for repressor only with forward and reverse transitions between repressor-bound and unbound states. The red rectangle is the repressor and green oval is the activator. Note that the four switching probabilities (P′A1, P′A2, P′R1 and P′R2) are different from the previous ones (PA1, PA2, PR1 and PR2) in the 3-state MCM. (b) Stochastic simulation for cell population at the steady state. (TIF) [file pone.0032376.s001.tif]

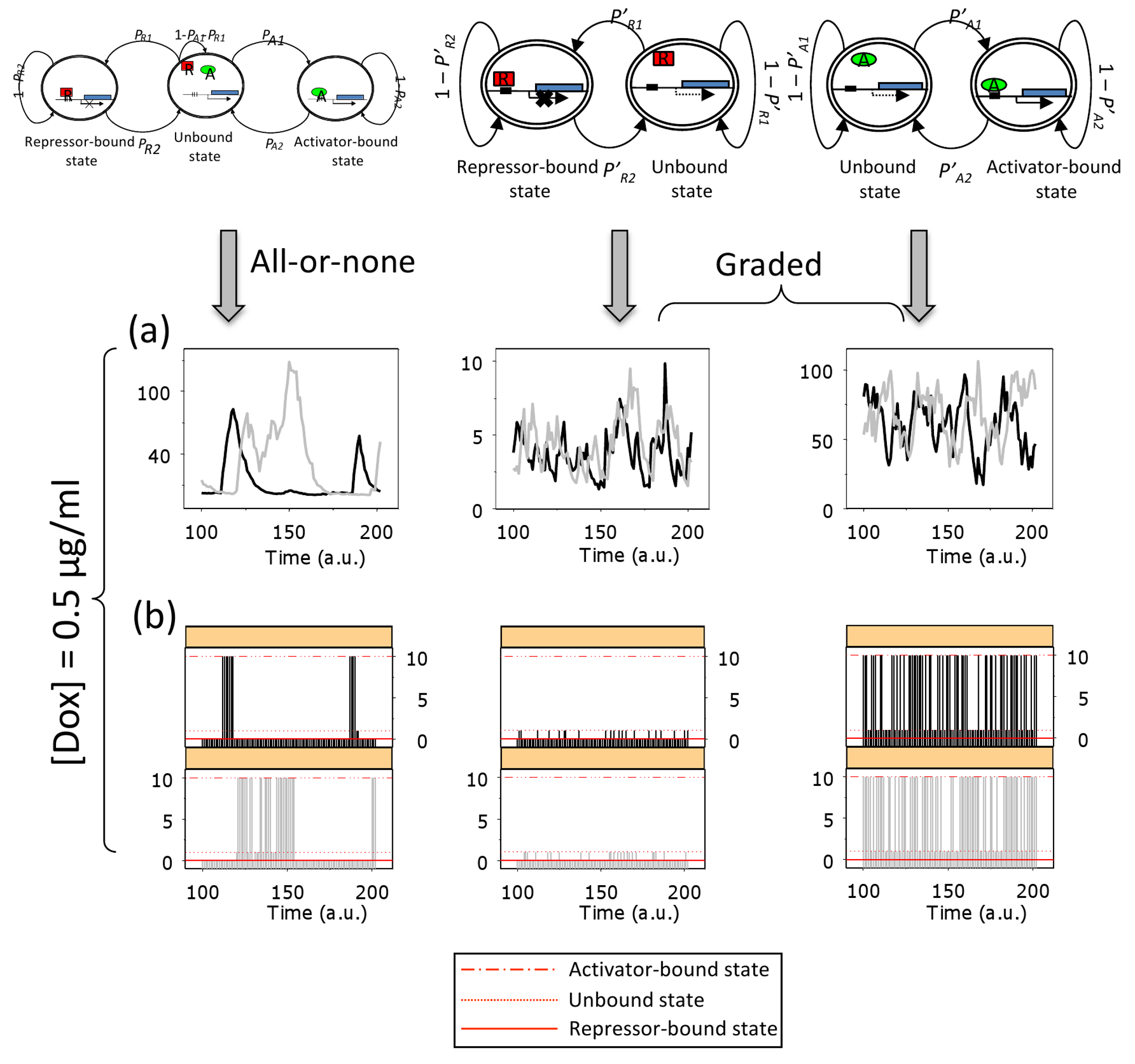

Supplement: Figure S2 — Dynamical fluctuations of simulated trajectories by MCM. (a) At the steady state and [dox] = 0.5 µg/ml, two time-series trajectories of two “single cell” stochastic simulations, randomly selected from 10,000 individual computer runs. (b) The corresponding telegraphs. Under this condition, the stochastic simulations of cell population exhibit switch-like patterns by the 3-state MCM (Figure 3b) or graded responses by the 2-state MCM (Figure S1). Three different types of horizontal red lines are drawn to denote the three states of transcription levels. (TIF) [file pone.0032376.s002.tif]

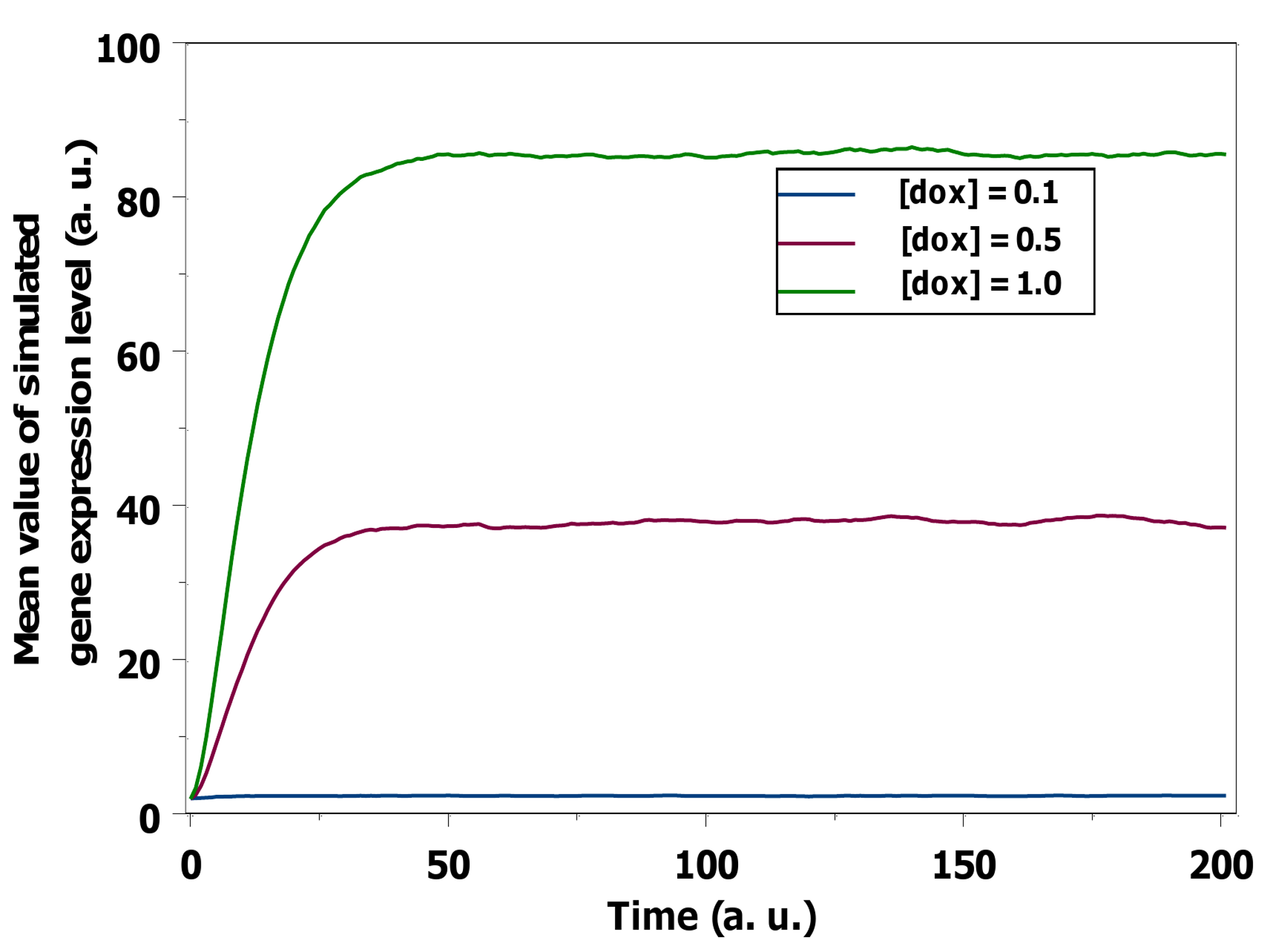

Supplement: Figure S3 — Averaged dynamical fluctuations of 10000 simulated trajectories by 3-state MCM. Simulation was carried out in three different [dox] conditions. The duration of this stochastic simulation is set from 0 to 201 time cycles. (TIF) [file pone.0032376.s003.tif]
