# Supplementary material for: Stochastic Modeling for the Expression of a Gene Regulated by Competing Transcription Factors
Source: PLoS One. 2012 Mar 14;7(3):e32376. doi: 10.1371/journal.pone.0032376 (PMC3303788; doi:10.1371/journal.pone.0032376)
Supplement: File S1 — Details about the modeling of experimental results reported in Rossi et al. (2000) by conventional method. (DOC) [file pone.0032376.s004.doc]

**Supplementary Information File S1: Details about the modeling of experimental results reported in Rossi et al. [1] by conventional method**

**Analytical solution of 3-state gene induction**

One common approach for modeling the expression of a single gene (Figure 1a) is the use of the Peccoud and Ycart model [2], in which changes of the gene state between “inactivity” and “activity” are the first-order rate constants rather than switching probabilities. In addition, Kim and O’Shea set up a thermodynamic model which is composed of 12 states of promoter activity mediated by two upstream activation sequences (UASs), Pho4 (TF), and nucleosome [3]. Similarly, 3-state gene induction by two competing TFs (Figure 1b) can be modeled by the time evolution of probability for the three states of the reporter gene into master equations [3,4].

$\begin{matrix} \frac{dP_{\text{Rep}}}{dt}=P_{R1}P_{\text{Unb}}-P_{R2}P_{\text{Rep}} \\ \frac{dP_{\text{Unb}}}{dt}=P_{R2}P_{\text{Rep}}+P_{A2}P_{\text{Act}}-(P_{R1}+P_{A1})P_{\text{Unb}} \\ \frac{dP_{\text{Act}}}{dt}=P_{A1}P_{\text{Uub}}-P_{A2}P_{\text{Act}} \end{matrix}$ **(S1)**

, where *P_Rep_*, *P_Act_*, and *P_Unb_* are the probabilities of the reporter gene to be bound by repressor, activator, and none of them, respectively. *P_A1_*, *P_A2_*, *P_R1_* and *P_R2_*, the same symbols with switching probabilities in the 3-state MCM, denote the first order rate constants for dynamical switching between repressor-bound, unbound and activator-bound promoter states. When the gene expression system reaches equilibrium, we can assume that the equation (Eq. **S1**) to equals zero and then solve for the analytical solution of steady-state probabilities (*P^s^*_Rep_, *P^s^*_Act_ and *P^s^*_Unb_):

$\begin{matrix} P_{\text{Rep}}^{s}=\frac{P_{A2}P_{R1}}{P_{A2}P_{R1}+P_{R2}P_{A2}+P_{R2}P_{A1}} \\ P_{\text{Unb}}^{s}=\frac{P_{R2}P_{A2}}{P_{A2}P_{R1}+P_{R2}P_{A2}+P_{R2}P_{A1}} \\ P_{\text{Act}}^{s}=\frac{P_{R2}P_{A1}}{P_{A2}P_{R1}+P_{R2}P_{A2}+P_{R2}P_{A1}} \end{matrix}$ **(S2)**

**Parameter estimation for Hill function**

The steady-state probability of the activator-bound state (*P^s^*_Act_) is presumably equal to the fraction of transcriptionally active promoters poised to produce mRNA. The gene induction, mediated by competing TFs (A + R), can be mathematically expressed in the form of *P^s^*_Act_. To obtain the dynamics of this gene induction, we have to estimate the parameter values of these four rate constants from the dose-response gene induction regulated by either activator only (A) or repressor only (R). Therefore, according to the assumption proposed in the main text (figure 2 and Eq. **3**) for the parameter estimation regarding the 3-state MCM, we are able to make the following expression:

$\begin{matrix} \frac{P_{\text{Act}}^{s}}{P_{\text{Act}}^{s}+P_{\text{Unb}}^{s}}=\frac{P_{A1}}{P_{A2}+P_{A1}}\approx OBS_{\text{Act}}=\frac{[dox]^{1.6}}{0.4^{1.6}+[dox]^{1.6}} \\ \frac{P_{\text{Unb}}^{s}}{P_{\text{Unb}}^{s}+P_{\text{Rep}}^{s}}=\frac{P_{R2}}{P_{R1}+P_{R2}}\approx OBS_{\text{Rep}}=\frac{[dox]^{1.8}}{0.8^{1.8}+[dox]^{1.8}} \end{matrix}$,

where the numerical values of the Hill coefficients and the half-effective [dox] are adopted from Figure 3c. Since the six parameters of the thermodynamic model were non-dimensionalized [3] to obtain the analytical expression which subsequently fit with dose-response profiles in the yeast *PHO5* gene expression system, it is reasonable to directly assign four switching rate constants with respect to the observed dose-response dynamics: *P_A1_* = [*dox*]^1.6^, *P_A2_* = 0.4^1.6^, *P_R2_* = [*dox*]^1.8^ and *P_R1_* = 0.8^1.8^. By plugging these parameter values into the analytical solution of *P^s^_AR_* (Eq. **S2**) and subsequently fitting them to Hill function, the Hill coefficient and effective concentration ([*dox*]) for gene induction were obtained and listed in the Table 1.

Without stochastic simulation, we are able to extract the Hill coefficient from dose-response experiments (A + R) using conventional methods. When compared with the observed Hill coefficient in the experiments by Rossi et al., the 3-state MCM has a closer estimation than that the value predicted by this conventional approach.

**Reference**

1. Rossi FMV, Kringstein AM, Spicher A, Guicherit OM, Blau HM (2000) Transcriptional control: rheostat converted to on/off switch. Mol Cell 6: 723-728.

2. Peccoud JY, B. (1995) Markovian modelling of gene product synthesis. Theor Popul Biol 48: 13.

3. Kim HD, O'Shea EK (2008) A quantitative model of transcription factor-activated gene expression. Nat Struct Mol Biol 15: 1192-1198.

4. Kepler TB, Elston TC (2001) Stochasticity in transcriptional regulation: origins, consequences, and mathematical representations. Biophysical Journal 81: 3116-3136.
