# Supplementary material for: Stochastic Modeling for the Expression of a Gene Regulated by Competing Transcription Factors
Source: PLoS One. 2012 Mar 14;7(3):e32376. doi: 10.1371/journal.pone.0032376 (PMC3303788; doi:10.1371/journal.pone.0032376)
Supplement: Table S1 — Estimated parameter values. (DOC) [file pone.0032376.s005.doc]

Table S1: Estimated parameter values

| **Parameter** | **2-state MCM** | **3-state MCM** |
| --- | --- | --- |
| Fitness | *ObjFunc2*^#^ = 0.0000 | *ObjFunc1*^#^ = 0.0308 |
| *H_A1_** | 1.6000 | 1.2944 |
| *K_A1_*** | 0.4000 | 0.6138 |
| *H_A2_** | 1.6000 | 1.3165 |
| *K_A2_*** | 0.4000 | 0.2636 |
| *H_R1_** | 1.8000 | 1.0114 |
| *K_R1_*** | 0.8000 | 0.0775 |
| *H_R2_** | 1.8000 | 1.0000 |
| *K_R2_*** | 0.8000 | 8.4781 |

* The range for estimating the Hill coefficient is from 1 to 5

** The range for estimating the effective [dox] is from 0 to 10

# The minimal values of objective functions (Eqs. **12** and **13)**
